# Unique considerations for the medical care of restrictive eating disorders in children and young adolescents

**DOI:** 10.1186/s40337-023-00759-2

**Published:** 2023-03-02

**Authors:** Anna B. Tanner

**Affiliations:** 1 Department of Pediatrics, Emory University, 30322 Atlanta, GA Georgia; 2 Dunwoody, Georgia

**Keywords:** Eating disorders, Children and young adolescents, Medical complications, Growth and development

## Abstract

**Background:**

The medical complications of eating disorders are often approached through an age-neutral lens. However, children and young adolescents may have unique medical complications related to the energy needs and timing of growth and development. Providers caring for patients in this vulnerable age range should understand how to identify, approach, and manage these potential age-related complications.

**Review:**

Evidence continues to accumulate that increasingly younger patients are being diagnosed with eating disorders. These children and young adolescents have significant risk for unique and potentially irreversible medical complications. Without early identification and treatment, restrictive eating disorders may negatively impact linear growth, bone development and brain maturation in children and young adolescents. Additionally, due to the energy needs of growth and development, unique considerations exist for the use of acute medical stabilization and the identification of patients at risk for refeeding syndrome with initial nutritional rehabilitation. This review presents an approach to the evaluation and management of children and young adolescents with eating disorders.

**Conclusion:**

Children and young adolescents with restrictive eating disorders may have unique medical complications related to the energy needs and timing of linear growth and pubertal development. Significant risk exists for irreversible medical complications of impaired growth, bone, and brain health. Increased awareness of the energy needs for growth and development may improve early recognition, appropriate intervention, and future outcomes for children and young adolescents with restrictive eating disorders.

## Abstract (summary)

Eating disorders are affecting increasingly younger patients, with ages of onset that now include children and young adolescents. This brings new risk, as children and young adolescents with eating disorders may develop unique medical complications related to the energy needs for the growth that should occur during this developmental stage. Inadequate energy intake during eating disorders in children and young adolescents can have long-term consequences including negative impacts on final height and bone and brain health. Children and young adolescents with eating disorders may present to care with acute concerns that require medical hospitalization and significant support to begin feeding again safely. Providers caring for children and young adolescents with eating disorders should be familiar with these unique complications and understand critical factors in the initial assessment, medical stabilization, and nutritional rehabilitation of children. The clock is ticking, and the goal of care of children and young adolescents with restrictive eating disorders should be to safely restore normal growth and puberty as quickly as possible.

## Background

Providers caring for the youngest patients in eating disorders care, specifically children and young adolescents, must recognize that critical differences exist in the medical evaluation and management of this population from that of older adolescents and adults. Current epidemiological trends and expanded recognition of eating disorders through new and revised diagnostic criteria have brought increasing numbers of younger patients to eating disorders care. Medical complications, due to restrictive behaviors, may present differently in children and young adolescents, who are pre-pubertal or peri-pubertal and have additional biological vulnerability due to the critical energy needs of growth and development.

Unique medical complications, related to linear growth, bone development and brain maturation, may be irreversible unless detected and addressed with effective early intervention. Heightened recognition of these developmentally based medical concerns and an increased understanding of current recommendations for acute medical management will improve outcomes and decrease morbidity and mortality associated with these illnesses in children and young adolescents with eating disorders.

## Current epidemiological trends of eating disorders in children and young adolescents

Eating disorders are common in children and adolescents, with increasingly younger patients affected. Estimates of the prevalence of all eating disorders in adolescents are 6–8% [1], although rates may be increasing as indicated by rising numbers of medical admissions for acute stabilization noted during the COVID-19 pandemic [2]. Recent studies show an increase in incidence of Anorexia Nervosa (AN) in younger patients, and a mean age of diagnosis of 12.2 years of age [3, 4]. This is consistent with prior studies that described an average age of onset of 12.3 years of age for AN [5]. In younger patients, males may represent a higher percentage of eating disorders patients than previously recognized, with one recent study showing that 24% of young patients presenting with AN were male [4].

Children and young adolescents can be affected by any eating disorder diagnosis. Revisions of diagnostic criteria in the Diagnostic and Statistical Manual of Mental Disorders (DSM-5) [6], have helped to clarify eating disorder diagnoses for many younger patients. New diagnoses, such as Atypical Anorexia Nervosa (AAN) and Avoidant/Restrictive Food Intake Disorder (ARFID) are seen frequently in children and young adolescents. Patients meeting criteria for ARFID comprised 14% of patients presenting for eating disorder care in one multicenter study [7]. In another study, one third of acute inpatient medical admissions for patients with restriction were patients with AAN [8]. Despite changes, substantial numbers of adolescents with disordered eating are still not receiving treatment [9].

Energy needs for growth and development create a period of nutritional vulnerability [10], which may produce a disparate medical impact in younger patients with restrictive eating disorders. Although all eating disorder diagnoses can be seen in children and young adolescents, the medical complications of restrictive eating disorders may generate urgent and potentially irreversible medical complications. Challenges exist in identifying and addressing unique medical complications for young patients with any restrictive behaviors, but predominantly those with AN, AAN and ARFID diagnoses. For children and young adolescents, unique medical complications related to restriction prevail and will be the primary focus of the remainder of this review.

## The initial medical evaluation in children and young adolescents

Treating increasingly younger patients with restrictive eating disorders brings new challenges in illness detection and medical management, as well as new risk due to the considerable energy needs and potential for missed opportunities for normal growth and development. Childhood and adolescence are a time-limited window for linear growth, bone development and brain maturation. When faced with starvation, growth failure and stunting may result [11]. Children should be growing, and adolescents should be in puberty. When restrictive eating disorders disrupt these critical developmental tasks, irreversible medical complications may result.

In adults and older adolescents, growth and puberty are finished, and patients with restriction may present overtly with weight loss accompanied by laboratory or vital sign abnormalities, amenorrhea or symptoms of low testosterone, and demonstrate medical compromise from starvation across organ systems. However, in children and young adolescents, growth and puberty are not complete. With eating disorders in prepubertal and peripubertal children and adolescents, growth retardation and pubertal delay may occur [12]. Medical evaluations must include discerning assessments of growth and puberty, and not rely solely on detecting overt weight loss, abnormal laboratory values, abnormal vital signs, amenorrhea or other symptoms of low hormone levels, to facilitate early detection and early intervention in younger eating disorders patients.

As in the initial medical evaluations in older adolescents and adults, a thoughtful and restrained rule out of other primary medical and psychiatric issues is prudent. However, providers must remember that extensive evaluations to rule out other causes may inadvertently delay initiation of care, resulting in increased severity of medical concerns. Anecdotally, these delays may also increase reluctance of patients and families to accept an eating disorders diagnosis when it is finally offered. Diagnosing younger patients with eating disorders can be difficult, as the link between eating disorders behaviors and presenting medical concerns may be less obvious. Restricted intake can be surreptitious and may result in lack of expected weight gain rather than obvious weight loss [13]. In younger patients, medical concerns such as constipation, headaches or dizziness may present before weight or intake changes are noted. Thus, medical concerns may inadvertently be perceived as the cause of the weight loss and restricted intake.

Per recommendations from the American Academy of Pediatrics (AAP), the initial medical evaluation of all children and young adolescents with known or suspected eating disorders should include an evaluation of growth parameters [12]. In addition to vital signs and laboratory studies, in young patients it is also useful to collect height and weight, determine pubertal status, review growth charts, and calculate mid-parental height (MPH). Current height can be compared to genetic potential using MPH, which may be calculated with the following formula:

- For biologic females (Mother’s height (in) + Father’s height (in) − 5)/2
  - Or (Mother’s height (cm) + Father's height (cm) − 13)/2
- For biologic males (Mother’s height (in) + Father's height (in) + 5)/2
  - Or (Mother’s height (cm) + Father's height (cm) + 13)/2
- Predicted range ± 3 in or 8.5 cm [14].

Growth charts should be reviewed and assessed in the context of growth patterns prior to illness onset. In malnourished children and young adolescents, a drop in weight percentile will be followed by a drop in height percentile on growth curves if energy deficiency is not amended. Shifts across two or more percentiles are uncommon in healthy growth [14]. The primary treatment goal for all young patients with restrictive eating disorders is to restore growth to prior linear growth channels and to maximize growth to genetic potential. Variances noted in growth curves may be used as a motivational interviewing tool in initial discussions with patients and parents.

Assessing severity of weight loss and malnutrition is crucial to adequately determine the impact of restriction and to appropriately direct initial steps for intervention, including identifying a need for weight restoration. For children and adolescents, weight restoration to support normal growth and development is an important goal of early treatment [15]. Although body mass index (BMI) is a frequently used tool in adult assessments, it is a less useful tool in younger patients when determining degree of malnutrition at presentation. The Society for Adolescent Health and Medicine (SAHM), proposes that malnutrition in children and adolescents be classified as mild, moderate, or severe, and factors to consider should include percent and rate of weight loss, percent median BMI (%mBMI), and BMI Z-score. For children and adolescents, SAHM proposes classifying severe malnutrition as %mBMI < 70%, BMI Z-score ≤ − 3, loss of more than 10% of body mass, or rapid weight loss (defined as > 5% in month, > 7.5% in 3 months, > 10% in 6 months or > 20% in one year) [15].

In children and young adolescents, changes in BMI Z-scores may be a useful indicator of malnutrition. Changes in BMI Z-scores may be used to evaluate both total weight loss and recency of weight loss, which have been shown to predict risk of medical complications in children and young adolescents.[8]. In addition, providers must remember that children and young adolescents may have decreased BMI Z-scores without any overt weight loss. BMI Z-scores will decrease when growing children fail to gain adequate weight while still increasing linear height. Constant mindfulness of normal growth and pubertal development, as well as use of age-appropriate markers of malnutrition are critical in the appropriate initial assessment of children and young adolescents with restrictive eating disorders [15].

## Impact of restrictive eating disorders on linear growth

The potential for eating disorders to disrupt linear growth in children and adolescents has been well described [13, 16]. Detection of deceleration of linear growth has been identified as a critical factor in the early identification of eating disorders in younger patients [13]. Supporting normal growth is a critical goal of nutritional rehabilitation [15]. Progression to skeletal maturity may limit potential adult height if weight restoration is delayed. Final adult heights shorter than that predicted by mid-parental height have been documented in adolescents with anorexia nervosa [17, 18]. Attention to growth in restrictive eating disorders is important, and weight restoration must occur to limit the final impact on adult linear height.

Children should be growing, and for young patients, growth curve deviations may alert providers that a restrictive eating disorder is present before parental suspicion is aroused. One study demonstrated growth curve deviations in 48% of adolescent eating disorders patients an average of 9.7 months before parents reported symptoms. Patients with slower weight loss were more likely to have symptoms noted after growth chart deviation had occurred, compared to patients with more acute weight loss [13]. Astute attention to growth curves may provide earlier detection of eating disorders in children and young adolescents and limit impact on final height.

Recent reviews demonstrate that catch-up growth may be suboptimal and may not be seen in all young patients with restrictive eating disorders. In a systematic review and meta-analysis of 27 studies, patients presenting at a younger age or with longer duration of illness were at greater risk for growth delay [16]. The authors emphasize that attention to growth “is important, especially in the young.”

Energy insufficiency will have a greater effect on growth when it is prolonged or occurs during times of rapid growth potential. Take off growth for biologic females occurs at age 9, with peak height velocity occurring at age 11, and for biologic males, these events occur 2 years later. The growth spurt accounts for 17–18% of final adult height [14]. When energy insufficiency occurs during these time limited developmental windows, final linear growth is impacted, as the opportunity to gain linear height is lost.

Goals for catch-up growth must be evaluated against genetic potential, by assessment of parental height, as well as growth curves [15]. Both male and female adolescents with AN are at risk for final adult heights lower than predicted by mid-parental target height [17, 18]. Poor catch-up growth may be an increasing concern as more patients present at younger ages. The AAP highlights that younger patients are at risk for greater and more permanent effects on growth [12]. Growth potential is lost as patients age. Weight restoration to support individual pre-morbid trajectories of linear growth to prior growth channels and to genetic potential is an important primary medical goal in all young patients presenting with restrictive eating disorders [15].

## Impact of restrictive eating disorders on bone development

Restrictive eating disorders have been identified as a risk factor for decreased bone mineral density (BMD) in adolescents. Peak bone mass is acquired during healthy adolescence, thus placing young patients with restrictive eating disorders at unique risk for poor bone health. Reductions in BMD in adolescents with AN have been well demonstrated [19]. Additionally, no significant sex differences in bone deficits are seen between male and female adolescents with AN, when controlling for age, duration of illness and %mBMI [20]. Impaired bone health has also been demonstrated in underweight adolescents with ARFID [21]. In adolescents with AAN, disordered bone health has also been described [22]; however additional studies suggest that a prior history of overweight may be associated with higher BMD Z-scores than patients with AN [23]. For adolescent athletes, weight bearing exercise and team sports may have some protective effect on BMD, however any benefits of exercise must be balanced against other risks in the management of the eating disorder [24].

In contrast to adult patients with AN, where decreases in BMD are due to both decreased formation and increased resorption, low bone density in young patients with anorexia nervosa primarily represents a missed opportunity for bone deposition. Significant bone mass (40–60%) is acquired in adolescence and multiple factors including low body weight, estrogen and testosterone deficits, GH resistance, low IGF-1 levels, and elevated cortisol levels may contribute to low bone mass [25]. In a longitudinal study, persistent negative effects on bone health were demonstrated in females with AN as adolescents despite recovery in body weight and BMI [26]. The development of AN during the adolescent years interrupts a critical window for bone mass acquisition. Understanding the potentially irreversible impact of restrictive eating disorders on bone health may serve as a “wake up call” for patients and parents [27]. Providers caring for children and young adolescents must convey that risk exists for poor bone density outcomes when restrictive eating disorders remain unchecked.

Sex hormones have a significant impact on bone health and are often impacted in restrictive eating disorders [25]. Adolescents should be in puberty, and appropriate assessment of pubertal onset and current sexual maturity in adolescents with restrictive eating disorders is a critical task [15]. Functional hypogonadism may occur in patients with poor nutrition and anorexia nervosa [28]. Biologic female adolescents typically have the onset of puberty a year before biologic males [28], with menarche between 12 and 12.5 years of age [29]. An assessment for other pubertal milestones, such as thelarche and pubarche [29], as well as a menarchal history should be collected in all biologic females at presentation. Biologic males typically begin puberty between ages 9 and 14, and will have milestones that include testicular enlargement, penile length increase, and development of pubic hair. Facial hair and voice deepening are later changes [29]. Providers may document physical exam findings of puberty by using a Sexual Maturity Rating (SMR) [29].

Providers should complete a thorough pubertal history on all eating disorder patients and complete an assessment of current sexual maturity [15]. In older adolescents and adults, commonly recognized symptoms of amenorrhea or decreased libido may indicate decreased estrogen or testosterone levels. In children and younger adolescents, malnutrition may present only as delayed or interrupted puberty, or later age of menarche [29], which may generate less explicit concern. This may inadvertently place younger adolescents at higher risk for poor bone health, especially adolescents in earlier stages of puberty, those with more significant pubertal delay, and those with longer pubertal suppression. In one study highlighting the risk of early onset AN, (AN onset prior to age 14 years old), patients with the most pubertal delay were found to have greatest negative impact on bone mineral content [30].

Updated guidelines for assessing bone health in adolescents with restrictive eating disorders are lacking. A recent international panel on child and adolescent bone health continued to define the need for dual energy X-ray absorptiometry (DXA) for patients with AN in terms of amenorrhea, recommending it for all patients with AN and amenorrhea for more than 6 months [31]. The AAP highlights that reduced BMD is related to malnutrition, as well as duration of amenorrhea in girls and low testosterone in boys. However, the AAP recommendations on when providers should evaluate bone health by DXA defers to children with clinically significant fractures and patients with medical conditions that place them at increased risk of fractures [25]. Among providers caring for adolescents with eating disorders, the lack of updated consensus guidelines has led to use of clinical factors in the decision-making process for young patients with restrictive eating disorders. Although one study found no difference in the rates of obtaining DXA scans for male and female patients, different clinical factors were identified in the decision-making process. Medical providers in this study used history of fractures, degree of malnutrition and duration of illness when deciding to order DXA scans for male patients and amenorrhea, history of fractures and duration of illness for female patients [32].

Recommendations from SAHM review that low bone mineral density occurs in adolescent patients with AN and that the optimal management remains unresolved [33]. Oral combined estrogen-progestin has not been found to increase BMD and is not recommended in adolescent females. Bisphosphonates are not currently recommended for adolescents with eating disorders and decreased BMD. One study on the use of transdermal estrogen did demonstrate increased spine and hip BMD scores [34]. Current SAHM guidelines emphasize weight restoration with spontaneous resumption of menses, optimization of calcium and vitamin D intake, and treatment of vitamin D deficiency if present [33]. For males, these recommendations could be extended to emphasize weight restoration with normalization of age-appropriate testosterone levels, optimization of calcium and vitamin D intake, and treatment of vitamin D deficiency if present.

## Impact of restrictive eating disorders on brain maturation

Although perhaps less apparent and more difficult to assess, restrictive eating disorders may have a deleterious impact on adolescent brain development. Part of normal childhood growth and adolescent development is critical brain growth and development. The child and young adolescent brain may be more vulnerable to the impact of starvation seen in patients with restrictive illnesses such as anorexia nervosa. Functional and structural changes, including changes to grey and white matter, have been observed in the brains of adolescents with eating disorders [35].

In adolescents with AN, loss of grey matter has been shown to be greater than that in adults. While these changes have been demonstrated to improve in adults with weight restoration, long-term studies demonstrating improvement in adolescents have been lacking [36]. Grey matter reduction in adolescents may be due to nutritional deficiencies resulting in neuronal apoptosis. Additionally, malnutrition may modify the process of dendritic pruning, a normal part of brain development in the maturing adolescent brain. These changes be an attempt to save energy during starvation and may explain the greater grey matter losses observed in adolescents. In patients with a diagnosis of AAN, no volumetric differences were found leading some researchers to hypothesize that a critical BMI threshold exists below which gray matter loss becomes apparent [35].

Additional studies evaluating white matter in adolescents with AN have found differences in microstructure by diffusion-weighted imaging, consistent with changes in myelination. These changes have been shown to potentially reverse with short-term weight restoration [37]. Similar changes to white matter have not been demonstrated in adolescents with AAN, further supporting the association between severe weight loss and alterations in white matter [38].

The dendritic pruning and increased myelin production that occur during healthy adolescence are associated with development of abstract thinking and executive functions. Cognition and reward pathways evolve during adolescence under the influence of reproductive and stress hormones. With starvation, brain changes occur over time and individuals may remain stuck in the adolescent brain and become more resistant to change. Early intervention for anorexia nervosa in children and young adolescents may mitigate the impact of starvation on the developing brain and minimize the duration of impact on neurodevelopment [39].

## Medical complications in children and young adolescents with restrictive eating disorders

Every organ system may be impacted by starvation in children and young adolescent patients with restrictive eating disorders. Children and adolescents are at risk for significant neurologic, cardiovascular, gastrointestinal, hematologic, and endocrine complications related to dietary restriction or weight loss [12], in addition to the unique concerns related to linear growth, bone development and brain maturation discussed previously.

Rate and percentage weight loss are important risk factors for many of the observed medical complications in children and adolescents. Renal impairment was shown in 33% of adolescents hospitalized for medical instability due to AN and AAN in one study. Patients presenting with more rapid weight loss were more likely to have renal impairment, suggested by a decreased estimated glomerular filtration rate (eGFR) [40]. In another study, elevated lipase levels, without evidence of pancreatitis, were identified in 34% of adolescent patients with AN or AAN presenting for inpatient treatment. Higher percentage of weight loss and lower %mBMI at presentation were associated with higher lipase levels [41].

Weight suppression, defined as the difference between premorbid and presentation weight, has been studied as a factor in medical complications in adolescent patients with AAN [42]. Total weight loss, as well as recency of weight loss, have been shown to be better predictors of many medical complications than presentation weight [8]. Similar rates of hypophosphatemia [43], bradycardia and orthostatic instability have been described in hospitalized adolescents with AN and AAN [44]. One study of adolescents with AN and AAN showed specifically that lower heart rate was associated with faster weight loss, and lower serum phosphorous levels were associated with greater amounts and longer durations of weight loss [42]. Despite these significant medical concerns, recent studies demonstrate decreased referrals of patients with AAN to eating disorder specific care [45]. and lower odds of adolescent patients with AAN receiving inpatient medical care [46]. Guidelines for assessing medical severity should continue to incorporate weight suppression to improve early detection and minimize medical complications.

Biologic male adolescents with restrictive eating disorders are not spared significant medical complications and may also face barriers in accessing adequate medical care. In one study, 39% of adolescent males had bradycardia that met SAHM hospital admission criteria at the time of presentation [47]. Progression to severe cardiovascular compromise may occur prior to eating disorder diagnosis. Recent studies have demonstrated that hospitalized adolescent males may also have increased medical needs once admitted for initial refeeding, including higher estimated energy requirements, higher caloric needs at discharge, longer admissions, and greater weight changes during medical stabilization. Associated factors included lower admission weight and lower admission heart rate, indicating increased medical severity by the time the eating disorder was identified [48]. Once an eating disorder is identified in an adolescent male, providers may be also less likely to screen for hypothalamic–pituitary–gonadal (HPG) axis suppression, with less frequent assessment of SMR, libido, or laboratory studies in males [49]. Guidelines for assessing medical severity and risk may need to be revised and adapted to adequately assess male adolescents with restrictive eating disorders, to improve earlier detection, and minimize medical complications.

Severe medical complications in children and adolescents with ARFID have been well documented and include significant eye disorders related to vitamin A deficiency, scurvy related to vitamin C deficiency, and anemia related to iron deficiency [50, 51]. Children and adolescent patients with ARFID have also been shown to have lower intake of vitamins K and B12 related to limited vegetable and protein intake [52], and deficiencies in thiamine and vitamin D [51]. Failure to make appropriate gains in weight and height during childhood and adolescence are known presentations [53], and weight loss and faltering growth are considered defining characteristics of ARFID [54]. However, significant nutritional deficiencies may be present even in ARFID patients that are not underweight and should not be missed [51]. Recent studies have shown similar medical comorbidities and endocrine conditions may exist in patients with AN and in patients with low weight ARFID, leading to calls from researchers to treat low weight ARFID as intensively as AN [55]. However, low intake may be chronic in patients with ARFID and associated with less bradycardia and less amenorrhea than in children and adolescents with AN and AAN, leading to less admissions for acute medical stabilization [56]. Guidelines for assessing medical severity may need to be revised and adapted with these findings in mind, to adequately assess children and adolescents with ARFID, to improve earlier detection, and to minimize medical complications.

Special considerations must be taken for the management of adolescents with disordered eating and gender identity concerns. Studies have shown higher prevalence of some eating disorder behaviors in gender nonconforming adolescents, in particular fasting in gender nonconforming males [57]. Due to concerns for potential restriction, screening for malnutrition in transgender youth has been advocated. For children and young adolescents with growth potential, specific challenges exist for evaluating and monitoring growth in the context of both disordered eating and gender transitioning. Consensus recommendations for monitoring growth in transgender individuals are lacking and adapted growth curves currently do not exist [58]. SAHM states that determination of weight goals in transgender adolescents “is particularly challenging” [15]. A recent case series reporting disordered eating among transgender youth with autism spectrum disorder highlights that care for these patients may be complex and require an individually tailored approach [59]. Current recommendations for adult transwomen and transmen identify low body weight as a risk factor for decreased bone health [60], adding further complexity and importance to identify standards for setting weight goals and monitoring growth in this population. Patients may need multidisciplinary teams for both gender-affirming care and eating disorder specific care and an individually tailored approach, especially when growth is a concern [59]. Once again, current guidelines to assess medical severity may need to be revised and adapted to adequately assess children and adolescents with eating disorders and gender concerns, to improve earlier detection and minimize medical complications.

## Acute medical stabilization in children and adolescents with eating disorders

At initial presentation, all patients with a known or suspected eating disorder must undergo an evaluation for medical stability and factors supporting acute medical hospitalization of children and adolescents are well established [15]. Children and young adolescents may be weight suppressed without overt weight loss and may become medically unstable rapidly due to the energy demands of growth and development. At presentation, patients may have depressed vital signs, significant electrolyte disturbances and electrocardiogram (ECG) abnormalities that necessitate admission for acute medical stabilization. Cardiovascular complications, including bradycardia, hypotension, and orthostatic vital signs changes, are frequently used indicators of need for medical stabilization in restrictive eating disorders in children and young adolescents. Guidelines from SAHM have provided criteria for inpatient admission for acute medical stabilization in children and adolescent [15].

Updated criteria from SAHM reinforce past recommendations for acute medical stabilization for children and young adolescents for concerns requiring immediate medical intervention or monitoring, including electrolyte disturbances, electrocardiogram abnormalities, and dehydration [15]. Vital sign abnormalities that support hospitalization include severe bradycardia (HR < 50 BPM daytime), hypotension (BP < 90/45 mm Hg), hypothermia (body temperature < 96 °F, 35.6 °C), or orthostatic vital sign changes (defined as sustained increase in HR > 40 bpm in adolescents under age 19 years or sustained decrease in blood pressure of > 20 mm Hg systolic or > 10 mm Hg diastolic) [15].

Bradycardia is a commonly used marker of instability in restrictive eating disorders in children and young adolescents. Longer duration of bradycardia and longer lengths of stay have been associated with lower presenting heart rates in adolescents with AN [61]. Thus, the recommendation to admit pediatric and adolescent patients with a heart rate below 50 beats per minute for acute medical stabilization remains unchanged in the updated guidelines [15].

Orthostatic vital sign changes have also been frequently used as a determinate of need for acute medical stabilization in children and adolescents. A significant update in the most recent criteria from SAHM allows for increased orthostatic heart rate and blood pressure changes than previously recommended, permitting orthostatic changes of 40 beats per minute in patients under 19 years of age compared to the 20 beats per minute that was included in all prior consensus statements [15]. This is consistent with current criteria for postural orthostatic tachycardia syndrome (POTS), that defines orthostatic tachycardia as an increase in heart rate of 40 beats per minute within 10 min of standing for patients aged 12–18 [62]. These newer criteria provide more latitude in following children and young adolescents without other acute medical concerns in an outpatient setting. However, once children and adolescents meet medical criteria for acute stabilization, providers should act swiftly due to provide acute medical support.

## Current practices for the detection and prevention of the medical complications of refeeding

Detecting and mitigating risk of medical complications during initial nutritional rehabilitation is a critical component of medical stabilization in children and young adolescents and requires access to a multidisciplinary team that includes experienced medical, nutrition, nursing and mental health teams, and standardized refeeding protocols [15]. Weight loss, BMI, and absolute oral intake have been shown to be less useful in determining refeeding risk in children and young adolescents with restrictive eating disorders. Proposed factors to better identify risk of refeeding syndrome in children and adolescents with restrictive eating disorders from the American Society of Parenteral and Enteral Nutrition (ASPEN) Consensus Recommendations include changes in BMI-for-age z-score, weight loss defined as less weight gain than expected, and less energy or protein intake than estimated need [63]. Any growing child or adolescent with a BMI z-score of − 3 or greater that is a change from baseline, who has weight loss defined as less than 25% of the norm for expected weight gain, or who has 7 consecutive days or more with protein or energy intake less than < 75% of estimated needs meets criteria for significant risk of refeeding syndrome by the ASPEN guidelines.

For adolescent patients hospitalized for acute medical complications of starvation, recent studies have shown that higher caloric intake during initial refeeding may restore medical stability and shorten hospital stays [64], without increasing rates of medical readmission or decreasing rates of clinical remission [65]. Additional studies continue to demonstrate the safety of higher calorie refeeding protocols in hospitalized adolescents, and that these protocols can lead to shorter lengths of stay without significant complications such as electrolyte issues, cardiac complications, or ICU admissions [66]. More conservative approaches that limit initial caloric intake may inadvertently result in slower weight gain and lengthen hospital stays [64]. One recent review of management guidelines for mitigating risk of refeeding in hospitalized malnourished adolescents concluded that higher initial caloric intake is supported by the evidence. These reviewers also propose the use of systemic phosphorous supplementation for adolescent patients with low phosphorous levels or those at high risk of refeeding syndrome [67]. Newer studies evaluating additional aspects of refeeding including the nutritional content of initial feeds are ongoing. One recent study demonstrated benefits of initial enteral feeds with lower carbohydrate and higher fat content to decrease the risk of hypophosphatemia [68].

The new position statement from SAHM on refeeding hypophosphatemia in hospitalized adolescents with anorexia nervosa states that refeeding hypophosphatemia correlates with the degree of malnutrition regardless of presentation weight and that higher calorie refeeding does not increase rates of hypophosphatemia, compared to traditional slower approaches to refeeding. These authors also state that additional research is still needed to identify risk factors for refeeding hypophosphatemia, the optimal nutritional content and delivery methods of initial feeds, and best strategies for electrolyte replacement [69]. Future research on the detection and prevention of refeeding syndrome in children and young adolescents is critical to allow for timely recovery when malnutrition from restriction has led to significant medical complications, including delays in growth and development.

## Conclusion

Ongoing efforts are needed to highlight the unique medical concerns in the care of children and young adolescents with restrictive eating disorders. Deceleration of linear height, impaired bone deposition and compromised brain development are potentially irreversible medical complications that can be decreased through an age-aware approach to eating disorders medical care. As more patients present at ever younger ages with restrictive eating disorders, methodologies to properly address the initial assessment of growth and development status, severity of malnutrition, and degree of weight suppression will be critical. Ongoing research is needed on methods to deliver nutrition safely and effectively to compromised children and young adolescents with restrictive eating disorders. Future guidelines should include recommendations for male adolescents, gender non-conforming adolescents, and adolescents with a history of living in a larger body who present with restrictive eating disorders. Providers caring for children and young adolescents with known or suspected restrictive eating disorders must remember that growth and development are not complete and when approaching the medical complications of our youngest eating disorders patients, special attention must be taken.

## Data Availability

Not applicable.

## Abbreviations

AN: Anorexia nervosa
AAN: Atypical anorexia nervosa
AAP: American academy of pediatrics
ARFID: Avoidant/restrictive food intake disorder
ASPEN: American society of parenteral and enteral nutrition
BMD: Bone mineral density
BMI: Body mass index
DXA: Dual energy X-ray absorptiometry
ECG: Electrocardiogram
HPG: Hypothalamic–pituitary–gonadal
MPH: Mid-parental height
POTS: Postural orthostatic tachycardia syndrome
SAHM: Society for adolescent health and medicine
SMR: Sexual maturity rating

## References

[1] Galmiche M, Déchelotte P, Lambert G, Tavolacci MP (2019). Prevalence of eating disorders over the 2000–2018 period: a systematic literature review. Am J Clin Nutr.

[2] Feldman MA, King CK, Vitale S, Denhardt B, Stroup S, Reese J (2022). The impact of COVID-19 on adolescents with eating disorders: increased need for medical stabilization and decreased access to care. Int J Eat Disord.

[3] van Eeden AE, van Hoeken D, Hoek HW (2021). Incidence, prevalence and mortality of anorexia nervosa and bulimia nervosa. Curr Opin Psychiatry.

[4] Morris A, Elliott E, Madden S (2022). Early-onset eating disorders in Australian children: a national surveillance study showing increased incidence. Int J Eat Disord.

[5] Swanson SA, Crow SJ, Le Grange D, Swendsen J, Merikangas KR (2011). Prevalence and correlates of eating disorders in adolescents: results from the national comorbidity survey replication adolescent supplement. Arch Gen Psychiatry.

[6] American Psychiatric Association. Diagnostic and Statistical Manual of Mental Disorders DSM-5. 5th ed. Arlington: American Psychiatric Association; 2013.

[7] Ornstein RM, Rosen DS, Mammel KA, Callahan ST, Forman S, Jay MS (2013). Distribution of eating disorders in children and adolescents using the proposed DSM-5 criteria for feeding and eating disorders. J Adolesc Health.

[8] Whitelaw M, Lee KJ, Gilbertson H, Sawyer SM (2018). Predictors of complications in anorexia nervosa and atypical anorexia nervosa: degree of underweight or extent and recency of weight loss?. J Adolesc Health.

[9] Sanzari CM, Liu RT (2019). Temporal trends in treatment utilization for disordered eating in US adolescents from 2004 through 2017: a nationally representative study. J Adolesc Health.

[10] Kumar MM, AlBuhairan F, Galagali P, Doctor AD, Weiss A, Keough L (2018). Addressing nutritional disorders in adolescents. J Adolesc Health.

[11] Golden NH, Nagata JM, Wade T (2015). Starvation in children, adolescents, and young adults: relevance to eating disorders. Encyclopedia of feeding and eating disorders.

[12] Hornberger LL, Lane MA, Lane M, Breuner CC, Alderman EM, Grubb LK (2021). Identification and management of eating disorders in children and adolescents. Pediatrics.

[13] Marion M, Lacroix S, Caquard M, Dreno L, Scherdel P, Guen CG (2020). Earlier diagnosis in anorexia nervosa: better watch growth charts!. J Eat Disord.

[14] Weintraub B (2010). Growth. Pediatr Rev.

[15] Golden NH, Katzman DK, Rome ES, Gaete V, Nagata JM, Ornstein RM (2022). Position paper of the society for adolescent health and medicine: medical management of restrictive eating disorders in adolescents and young adults. J Adolesc Health.

[16] Neale J, Pais SM, Nicholls D, Chapman S, Hudson LD (2020). What are the effects of restrictive eating disorders on growth and puberty and are effects permanent? A systematic review and meta-analysis. J Adolesc Health.

[17] Modan-Moses D, Yaroslavsky A, Pinhas-Hamiel O, Levy-Shraga Y, Kochavi B, Iron-Segev S (2021). Prospective longitudinal assessment of linear growth and adult height in female adolescents with anorexia nervosa. J Clin Endocrinol Metab.

[18] Modan-Moses D, Yaroslavsky A, Novikov I, Segev S, Toledano A, Miterany E (2003). Stunting of growth as a major feature of anorexia nervosa in male adolescents. Pediatrics.

[19] Misra M, Golden NH, Katzman DK (2016). State of the art systematic review of bone disease in anorexia nervosa. Int J Eat Disord.

[20] Nagata JM, Golden NH, Peebles R, Long J, Leonard MB, Chang AO (2017). Assessment of sex differences in bone deficits among adolescents with anorexia nervosa. Int J Eat Disord.

[21] Alberts Z, Fewtrell M, Nicholls DE, Biassoni L, Easty M, Hudson LD (2020). Bone mineral density in anorexia nervosa versus avoidant restrictive food intake disorder. Bone.

[22] Pehlivanturk-Kizilkan M, Akgul S, Derman O, Kanbur N (2021). Predictors of bone mineral density in adolescents with atypical anorexia nervosa. J Bone Miner Metab.

[23] Nagata JM, Carlson JL, Golden NH, Long J, Murray SB, Peebles R (2019). Comparisons of bone density and body composition among adolescents with anorexia nervosa and atypical anorexia nervosa. Int J Eat Disord.

[24] Nagata JM, Carlson JL, Golden NH, Murray SB, Long J, Leonard MB, Peebles R (2019). Associations between exercise, bone mineral density, and body composition in adolescents with anorexia nervosa. Eat Weight Disord.

[25] Golden NH, Abrams SA (2014). Committee on Nutrition. Optimizing bone health in children and adolescents. Pediatrics.

[26] Mumford J, Kohn M, Briody J, Miskovic-Wheatley J, Madden S, Clarke S (2019). Long-term outcomes of adolescent anorexia nervosa on bone. J Adolesc Health.

[27] DiVasta AD, Gordon CM (2019). Long-term skeletal consequences of anorexia nervosa: a “wake up call”. J Adolesc Health.

[28] Howard SR (2021). Interpretation of reproductive hormones before, during and after the pubertal transition—identifying health and disordered puberty. Clin Endocrinol.

[29] Wolf RM, Long D (2016). Pubertal development. Pediatr Rev.

[30] Clarke J, Peyre H, Alison M, Bargiacchi A, Stordeur C, Boizeau P (2021). Abnormal bone mineral density and content in girls with early-onset anorexia nervosa. J Eat Disord.

[31] Galindo-Zavala R, Bou-Torrent R, Magallares-López B, Mir-Perelló C, Palmou-Fontana N, Sevilla-Pérez B (2020). Expert panel consensus recommendations for diagnosis and treatment of secondary osteoporosis in children. Pediatr Rheumatol.

[32] Nelson L, Carlson J, Halpern-Felsher B, Nagata J (2022). Assessing clinician comfort and screening practices for evaluating bone mineral density in adolescents and young adults with an eating disorder based on patient sex. J Adolesc Health.

[33] Golden NH, Katzman DK, Sawyer SM, Ornstein RM, Rome ES, Garber AK (2015). Update on the medical management of eating disorders in adolescents. J Adolesc Health.

[34] Misra M, Katzman D, Miller KK, Mendes N, Snelgrove D, Russell M (2011). Physiologic estrogen replacement increases bone density in adolescent girls with anorexia nervosa. J Bone Miner Res.

[35] Kappou K, Ntougia M, Kourtesi A, Panagouli E, Vlachopapadopoulou E, Michalacos S (2021). Neuroimaging findings in adolescents and young adults with anorexia nervosa: a systematic review. Children (Basel).

[36] Seitz J, Herpertz-Dahlmann B, Konrad K (2016). Brain morphological changes in adolescent and adult patients with anorexia nervosa. J Neural Transm.

[37] Griffiths KR, Martin Monzon B, Madden S, Kohn MR, Touyz S, Sachdev PS (2021). White matter microstructural differences in underweight adolescents with anorexia nervosa and a preliminary longitudinal investigation of change following short-term weight restoration. Eat Weight Disord.

[38] Olivo G, Swenne I, Zhukovsky C, Tuunainen AK, Saaid A, Salonen-Ros H (2019). Preserved white matter microstructure in adolescent patients with atypical anorexia nervosa. Int J Eat Disord.

[39] Mysliwiec R (2020). Neuroscience of adolescent anorexia nervosa: Implications for family-based treatment (FBT). Front Psychiatry.

[40] Downey AE, Cheng J, Adams SH, Buckelew SM, Kapphahn CJ, Machen VI (2022). Renal function in patients hospitalized with anorexia nervosa undergoing refeeding: findings from the study of refeeding to optimize inpatient gains. J Adolesc Health.

[41] Kiesow K, Shahid N, Park CC, Weaver L, Lantzouni E, Peebles R (2018). Pancreatic enzyme elevation in adolescents with eating disorders. J Adolesc Health.

[42] Garber AK, Cheng J, Accurso EC, Adams SH, Buckelew SM, Kapphahn CJ (2019). Weight loss and illness severity in adolescents with atypical anorexia nervosa. Pediatrics.

[43] Whitelaw M, Gilbertson H, Lee KJ, Sawyer SM (2014). Restrictive eating disorders among adolescent inpatients. Pediatrics.

[44] Sawyer SM, Whitelaw M, Le Grange D, Yeo M, Hughes EK (2016). Physical and psychological morbidity in adolescents with atypical anorexia nervosa. Pediatrics.

[45] Harrop EN, Mensinger JL, Moore M, Lindhorst T (2021). Restrictive eating disorders in higher weight persons: a systematic review of atypical anorexia nervosa prevalence and consecutive admission literature. Int J Eat Disord.

[46] Kennedy GA, Forman SF, Woods ER, Hergenroeder AC, Mammel KA, Fisher MM (2017). History of overweight/obesity as predictor of care received at 1-year follow-up in adolescents with anorexia nervosa or atypical anorexia nervosa. J Adolesc Health.

[47] Vo M, Lau J, Rubinstein M (2016). Eating disorders in adolescent and young adult males: presenting characteristics. J Adolesc Health.

[48] Nagata JM, Bojorquez-Ramirez P, Nguyen A, Ganson KT, Machen VI, Cattle CJ (2022). Sex differences in refeeding among hospitalized adolescents and young adults with eating disorders. Int J Eat Disord.

[49] Nelson LR, Halpern-Felsher BL, Nagata JM, Carlson JL (2021). Clinician practices assessing hypothalamic–pituitary–gonadal axis suppression in adolescents with an eating disorder. Int J Eat Disord.

[50] Yanagimoto Y, Ishizaki Y, Kaneko K (2020). Iron deficiency anemia, stunted growth, and developmental delay due to avoidant/restrictive food intake disorder by restricted eating in autism spectrum disorder. Biopsychosoc Med.

[51] Yule S, Wanik J, Holm EM, Bruder MB, Shanley E, Sherman CQ (2021). Nutritional deficiency disease secondary to ARFID symptoms associated with autism and the broad autism phenotype: a qualitative systematic review of case reports and case series. J Acad Nutr Diet.

[52] Harshman SG, Wons O, Rogers MS, Izquierdo AM, Holmes TM, Pulumo RL (2019). A diet high in processed foods, total carbohydrates and added sugars, and low in vegetables and protein is characteristic of youth with avoidant/restrictive food intake disorder. Nutrients.

[53] Brigham KS, Manzo LD, Eddy KT, Thomas JJ (2018). Evaluation and treatment of avoidant/restrictive food intake disorder (ARFID) in adolescents. Curr Pediatr Rep.

[54] Eddy KT, Harshman SG, Becker KR, Bern E, Bryant-Waugh R, Hilbert A (2019). Radcliffe ARFID workgroup: toward operationalization of research diagnostic criteria and directions for the field. Int J Eat Disord.

[55] Aulinas A, Marengi DA, Galbiati F, Asanza E, Slattery M, Mancuso CJ (2020). Medical comorbidities and endocrine dysfunction in low-weight females with avoidant/restrictive food intake disorder compared to anorexia nervosa and healthy controls. Int J Eat Disord.

[56] Keery H, LeMay-Russell S, Barnes TL, Eckhardt S, Peterson CB, Lesser J (2019). Attributes of children and adolescents with avoidant/restrictive food intake disorder. J Eat Disord.

[57] Gordon AR, Austin SB, Schultz J, Guss CE, Calzo JP, Wang ML (2021). Gender expression, peer victimization, and disordered weight-control behaviors among US high school students. J Adolesc Health.

[58] Moser C, Voss M, Jansen-Kraly K, Jacobson J (2017). Gender identity and malnutrition: so much remains unknown. J Adolesc Health.

[59] Pham A, Kasenic A, Hayden L, Inwards-Breland DJ, Sumerwell C, Twible H (2021). A case series on disordered eating among transgender youth with autism spectrum disorder. J Adolesc Health.

[60] Stevenson MO, Tangpricha V (2019). Osteoporosis and bone health in transgendered persons. Endocrinol Metab Clin North Am.

[61] Bako A, Yeo M, Sawyer SM, Hughes E (2019). How low can you go? The significance of bradycardia for acute clinical outcomes in hospitalised adolescents with anorexia nervosa. J Adolesc Health.

[62] Raj SR, Guzman JC, Harvey P, Richer L, Schondorf R, Seifer C (2020). Canadian cardiovascular society position statement on postural orthostatic tachycardia syndrome (POTS) and related disorders of chronic orthostatic intolerance. Can J Cardiol.

[63] da Silva JS, Seres DS, Sabino K, Adams SC, Berdahl GJ, Citty SW (2020). ASPEN consensus recommendations for refeeding syndrome. Nutr Clin Pract.

[64] Garber AK, Cheng J, Accurso EC, Adams SH, Buckelew SM, Kapphahn CJ (2021). Short-term outcomes of the study of refeeding to optimize inpatient gains for patients with anorexia nervosa: a multicenter randomized clinical trial. JAMA Pediatr.

[65] Golden NH, Cheng J, Kapphahn CJ, Buckelew SM, Machen VI, Kreiter A (2021). Higher-calorie refeeding in anorexia nervosa: 1-year outcomes from a randomized controlled trial. Pediatrics.

[66] Schlapfer L, Fujimoto A, Gettis M (2022). Impact of caloric prescriptions and degree of malnutrition on incidence of refeeding syndrome and clinical outcomes in patients with eating disorders: a retrospective review. Nutr Clin Pract.

[67] Proulx-Cabana S, Metras ME, Taddeo D, Jamoulle O, Frappier JY, Stheneur C (2022). To improve the initial inpatient management of adolescents admitted with severe anorexia nervosa: a narrative review and a convenient protocol. Nutrients.

[68] Parker EK, Flood V, Halaki M, Wearne C, Anderson G, Gomes L (2021). A standard enteral formula versus an iso-caloric lower carbohydrate/high fat enteral formula in the hospital management of adolescent and young adults admitted with anorexia nervosa: a randomised controlled trial. J Eat Disord.

[69] Katzman DK, Garber AK, Parker EK, Kohn M, Golden NH (2022). Position statement of the society for adolescent health and medicine: refeeding hypophosphatemia in hospitalized adolescents with anorexia nervosa. J Adolesc Health.

